# Trophic and symbiotic links between obligate-glacier water bears (Tardigrada) and cryoconite microorganisms

**DOI:** 10.1371/journal.pone.0262039

**Published:** 2022-01-12

**Authors:** Krzysztof Zawierucha, Artur Trzebny, Jakub Buda, Elizabeth Bagshaw, Andrea Franzetti, Miroslawa Dabert, Roberto Ambrosini

**Affiliations:** 1 Department of Animal Taxonomy and Ecology, Adam Mickiewicz University, Poznań, Poland; 2 Molecular Biology Techniques Laboratory, Faculty of Biology, Adam Mickiewicz University, Poznań, Poland; 3 School of Earth and Environmental Sciences, Cardiff University, Cardiff, United Kingdom; 4 Earth and Environmental Sciences Department, University of Milano-Bicocca, Milan, Italy; 5 Department of Environmental Science and Policy, University of Milan, Milan, Italy; University of Illinois Urbana-Champaign, UNITED STATES

## Abstract

Insights into biodiversity and trophic webs are important for understanding ecosystem functions. Although the surfaces of glaciers are one of the most productive and biologically diverse parts of the cryosphere, the links between top consumers, their diet and microbial communities are poorly understood. In this study, for the first time we investigated the relationships between bacteria, fungi and other microeukaryotes as they relate to tardigrades, microscopic metazoans that are top consumers in cryoconite, a biologically rich and productive biogenic sediment found on glacier surfaces. Using metabarcoding (16S rDNA for bacteria, ITS1 for fungi, and 18S rDNA for other microeukaryotes), we analyzed the microbial community structures of cryoconite and compared them with the community found in both fully fed and starved tardigrades. The community structure of each microbial group (bacteria, fungi, microeukaryotes) were similar within each host group (cryoconite, fully fed tardigrades and starved tardigrades), and differed significantly between groups, as indicated by redundancy analyses. The relative number of operational taxonomic units (ZOTUs, OTUs) and the Shannon index differed significantly between cryoconite and tardigrades. Species indicator analysis highlighted a group of microbial taxa typical of both fully fed and starved tardigrades (potential commensals), like the bacteria of the genera *Staphylococcus* and *Stenotrophomonas*, as well as a group of taxa typical of both cryoconite and fully fed tardigrades (likely part of the tardigrade diet; bacteria *Flavobacterium* sp., fungi *Preussia* sp., algae *Trebouxiophyceae* sp.). Tardigrades are consumers of bacteria, fungi and other microeukaryotes in cryoconite and, being hosts for diverse microbes, their presence can enrich the microbiome of glaciers.

## Introduction

Identification of trophic networks and relationships between animals with other biotic and abiotic components of an ecosystems is a crucial step towards understanding how an ecosystem functions, how it will respond to changes in supply resources or what services particular organisms play in the ecological community [1–3]. Microscopic commensal and parasitic species are known to play a role in ecosystem processes, but their contributions are poorly understood [2, 4]. Studies on microbial communities facilitate the understanding of the relationships between organisms and their internal and external environments, and are essential to gain insights into evolutionary processes [5–7]. Simple ecosystems are an ideal model for exploring host-environment interactions and microbiological processes, and an ideal example can be found in cryoconite holes on glacier surfaces.

Although glaciers cover ca. 10% of land surface, little attention has been given to understanding the relationships between microbial communities and higher trophic consumers in these cold ecosystems [8–10]. The most species-rich and productive ice surface habitats are cryoconite holes [11–13]. Cryoconite sediment is a mixture of mineral and organic matter, including psychrophilic autotrophic and heterotrophic microbes, with associated invertebrates that play the role of grazers [14–18]. The dark color of the cryoconite reduces ice albedo, influences ice melting and, in favorable conditions, determines the formation of cryoconite holes, which are water-filled depressions in the surface of ice. Thanks to the presence of cryoconite and of melted water, cryoconite holes function as biogeochemical ‘factories’ that strongly affect matter and energy flow on ice [17, 19–22]. Whilst the biogeochemical status and basic microbial community structure of cryoconite holes has been explored, still many knowledge gaps exist that limit our understanding of these unique ecosystems [11, 18]. In particular, we have very limited information on the potential food of grazers and on their microbiota [17]. We need to know what part of the supraglacial microbiome belongs exclusively to animal symbionts, or how food preferences of grazers shape supraglacial microbial communities [8–10]. For example, Murakami et al. [9] suggested that the gut bacteria-host association in the glacier stonefly *Andiperla willinki* contributes to both host nutrition and to material cycles in glacier environments.

One of the most common invertebrates inhabiting glaciers around the world are Tardigrada [18], a phylum also known as water bears [23]. The studies on diet and food preferences of limnoterrestrial tardigrades have revealed that tardigrades may be divided into various trophic groups [24, 25], albeit often they utilize the available food sources regardless of the feeding group [26]. Until now, only a few studies have focused on the diet and role of invertebrates, including tardigrades, in cryoconite trophic networks. Vonnahme et al. [27] found that Tardigrada and Rotifera densities do not show any significant negative correlation with microalgal (potential food) abundances on Svalbard glaciers, but found that most microalgae in cryoconite on Svalbard form large colonies, which may protect them against invertebrate grazing. Zawierucha et al. [17] and Buda et al. [22] did not find any clear trophic relationship between primary producers and grazers in cryoconite holes on the margin of the Greenland Ice Sheet (Russel Glacier) or on a maritime Antarctic glacier, respectively. In contrast, Jaromerska et al. [28], using stable isotope ratios, revealed different use of food sources by tardigrades and rotifers in High Arctic cryoconite holes, although the food sources were unidentified. These studies were not able to present robust and clear relationships between grazers, their food sources and other biota. Other approaches are therefore necessary, and we propose that the study of the microbiota associated with top consumers, particularly tardigrades, can identify the trophic webs that form in cryoconite microbial communities.

Information about glacial invertebrates, their microbiota and their potential food are scant. One notable exception are the studies by Murakami et al. [8–10] who used molecular approaches to investigate oligochaetes from maritime North American glaciers and plecopterans from South American ice fields. These studies revealed that glacial invertebrates host distinct bacterial communities, but their microbiome is strongly influenced by the environment. Some of the glacier bacteria could be used as food, while others were exploited by invertebrates as transient gut symbionts [8]. Moreover, particular groups of invertebrate microbiota could help in the digestion of ice algae, an abundant potential food source on the ice surface [9, 10]. Until now, only Vecchi et al. [29] fully characterized the microbiome of six limnoterrestrial tardigrade species and revealed that also in this case, the microbiota was mostly determined by the environment, but unique bacterial operational taxonomic units (OTUs) were identified in tardigrades.

**Fig 1:**
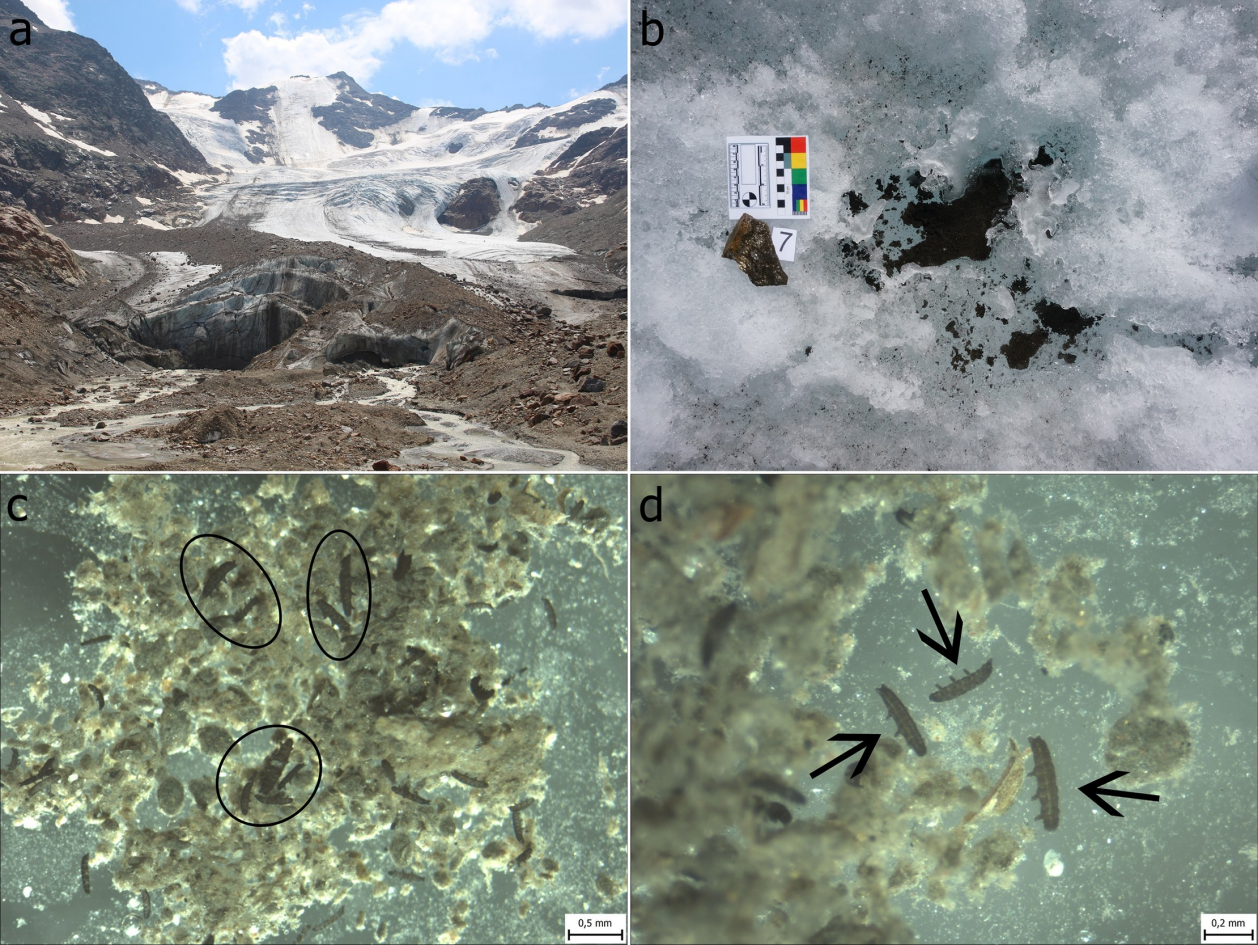
a) Forni Glacier during the ablation season. The approximate width of the glacier terminus is 240 meters (details on the recent evolution of glacier are provided in [35], phot. K. Zawierucha). b) cryoconite hole on Forni Glacier (scale bar on the picture = 8 cm), c, d) tardigrades, *Cryobiotus klebelsbergi* in cryoconite. Ellipses and arrows indicate tardigrades in cryoconite sediments.

The symbionts and parasites may affect different aspects of host fitness [30–34], which in harsh glacier ecosystems can be of crucial importance. In this study we used three genetic markers and next generation sequencing (NGS) to identify bacteria, fungi and other microeukaryotes in cryoconite and in microscopic metazoans (tardigrades) on Forni Glacier, one of the most extensively studied glacier in the Alps with well recognized bacterial and metazoans communities [20, 21, 35]. In particular, cryoconite ecosystems on Forni are dominated by one species of obligate glacier tardigrade, *Cryobiotus klebelsbergi* (Fig 1). Our aim was to understand whether tardigrades are consumers of specific biotic components in cryoconite, and whether they host unique microbial communities, different from those in cryoconite. To this end, we identified: i) the cryoconite microbiota (bacteria, fungi and other microeukaryotes); ii) the potential host-associated microbial community (tardigrade microbiome); and iii) tardigrade putative food sources.

## Material and methods

### Study site and model organism

Forni Glacier, in the Ortles–Cevedale group (Stelvio National Park, Central Italian Alps), is one of the largest Italian glaciers (Fig 1A). Its elevation ranges between 2600 and 3670 m a.s.l. [35]. Cryoconite was collected from the bottom of cryoconite holes, from puddles and from aggregations of cryoconite on the surface of the glacier in the ablation zone (Fig 1B) during a sampling campaign in July 2019. Sampling was conducted between 2700 and 2800 m a.s.l. The cryoconite was collected from many sites separated from each other by tens of meters to collect diverse subsamples that constitute extensive representation of tardigrade diet. Taking into account the regular mixing of cryoconite material due to rain or meltwater flow on the surface of Forni, the strategy of random and scattered sampling, then pooling all cryoconite into one sample, was to account for heterogeneity of the supraglacial (surface) material. Samples were collected with independent sterile disposable plastic Pasteur pipettes or a sterile (washed by alcohol) stainless spoon and transferred into sterile plastic bags, then frozen and transported to the laboratory at Adam Mickiewicz University, Poznań (Poland), where they were kept at -20°C until further analyses. All samples were properly collected and analyzed under the permission of local organization (Stelvio National Park, Italy) granted to R. Ambrosini and A. Franzetti.

*Cryobiotus klebelsbergi* (Fig 1C and 1D) is a typical glacier obligate tardigrade whose known distribution includes a few glaciers in the Alps [21, 36, 37]. Black pigmentation of *C*. *klebelsbergi* is proposed to protect against high UV irradiation of high mountain glaciers [38]. This species lives exclusively in cryoconite of cryoconite holes and it is absent in the proglacial field, mosses on moraines or freshwater reservoirs around the glacier [21]. Field and laboratory experiments confirmed *C*. *klebelsbergi* is a typical glacier obligate and temperature sensitive species [21].

### Animal extraction

In the laboratory, cryoconite samples were slowly melted at +4°C to avoid drastic changes of temperatures, which may negatively influence psychrophilic glacier organisms. The experimental design followed Murakami et al. [8] with some modifications. We divided tardigrades and cryoconite under sterilized stereomicroscopes in a sterile laminar flow chamber. Due to the sensitivity of *C*. *klebelsbergi* to high temperatures [21], we extracted tardigrades placing the samples on ice or ice packs. Tardigrades extracted directly from melted cryoconite were considered as fully fed (i.e. we assumed they were feeding on cryoconite just before freezing and after melting). Cryoconite without tardigrades and tardigrades alone extracted from cryoconite in the former step were put in separate Eppendorf tubes for further DNA analysis. Other tardigrades extracted from cryoconite were individually placed onto 24-well sterile plastic plates with one milliliter of miliQ water in each well. Plates were then secured with parafilm and tardigrades were kept without food at +1.5°C for three weeks. Before DNA extraction, the animals were vortexed in 1 ml of sterile water in a 1.5 ml Eppendorf tube for 1 min. After that, the tube was gently centrifuged to settle the animals, while the supernatant with potential contamination, coming from the external cuticle of animals or feces (potentially originating from birds flying over the glacier), was collected and used as a control. Each tardigrade was washed three times in miliQ water before DNA extraction. In total we prepared six replicates of cryoconite (1 ml of wet cryoconite per sample), four replicates of fully fed tardigrades and three replicates of starved tardigrades (each tardigrade sample contained 10 specimens, in total we used 70 specimens), seven control samples (one per each fully fed and starved tardigrades) and one sample of sterile water was used to detect contamination.

### DNA extraction and amplicon library generation

For DNA isolation we used a FastDNA SPIN Kit for Soil and followed the manufacturer’s instruction (http://dmoserv3.whoi.edu/data_docs/IODP_347/FastDNA_Spin_Kit_for_Soil.pdf). Eukaryotic and prokaryotic markers were based on fragments of nuclear small subunit rRNA gene (hereafter rDNA) covering hypervariable regions V9 (V9 18S rDNA) and V4 (V4 16S rDNA), respectively. Fungal DNA was detected by amplification of the internal transcribed spacer 1 (ITS1) (S1 Table in S1 File). All PCR primers for NGS sequencing used in this study (S1 Table in S1 File) were tailed at 5’ ends with dual-indexed Ion Torrent adapters for sequencing using the Ion Torrent system (Life Technologies, USA).

All DNA markers were amplified in two technical replications, each in a total volume of 10 μl containing Hot FIREPol DNA Polymerase, 0.25 μM of each tailed primer and 1 μl of template DNA. PCR program was as follows: 12 min at 95°C, followed by 35 cycles of 15 s at 95°C, 30 s at 50°C and 30 s at 72°C, with a final extension step at 72°C for 5 min. Negative control samples from blank extractions and blank PCR reactions were analyzed in the same way as test samples. For each PCR reaction, 3 μl were electrophoresed on a 1.5% agarose gel to check amplification efficiency. Amplicons corresponding to genetic markers were pooled, agarose gel-fractionated, and purified using 3% agarose gel electrophoresis and QIAquick Gel Extraction Kit (Qiagen, Germany) according to the manufacturer’s instructions (https://qiagen.com/us/resources/resourcedetail?id=95f10677-aa29-453d-a222-0e19f01ebe17&lang=en).

### Amplicon sequencing and bioinformatics

DNA concentration and fragment length distribution of libraries were established with the use of a High Sensitivity D1000 Screen Tape assay on a 2200 Tape Station system (Life Technologies, USA). Clonal template amplifications were performed using the Ion Torrent OT2 Kit (Life Technologies, USA) according to manufacturer’s instructions (https://tools.thermofisher.com/content/sfs/manuals/MAN0010850_Ion_540_OT2_UG.pdf).

Sequencing was carried out using Hi-Q View Sequencing Kit and Ion S5 system on an Ion 540 chip (Life Technologies, USA) according to the manufacturer’s instructions (https://tools.thermofisher.com/content/sfs/manuals/MAN0010850_Ion_540_OT2_UG.pdf).

Raw sequence data were pre-filtered by Ion Torrent Suite software version 5.10.1 (Life Technologies, USA) to remove polyclonal and low quality sequences. Further bioinformatic analysis was conducted using fastq data and custom workflow. Sequence reads shorter than 180-bp were removed from the dataset. Leading and trailing low-quality bases were removed using Trimmomatic version 0.39 [39]. Fastx Toolkit [40] was used to extract sequences with at least 50% of bases with a quality score ≥ 25. Quality filtered sequences were separated into individual combinations of indexes and trimmed at 5’- and 3’-ends to exclude PCR primers in Geneious R11.1.5. The singletons (< 10 reads) were removed using the -fastx_uniques and -sortbysize algorithms [41]. Chimeras were removed using the default settings in UCHIME2 version 4.2.40 [42] and SILVA database for ARB for small subunit ribosomal RNAs version 132 [43–45] as implemented in Geneious R11.1.5. Operational taxonomic units (OTU) were clustered from sequences whose abundance exceeded a threshold of 10 counts using the -cluster_otus algorithm implemented in USEARCH version 11.0.667 [41]. Additionally, prokaryotic sequences were denoised into zero-radius operational taxonomic units (ZOTUs) and subsequently a ZOTU table was constructed according to the -denoising steps [42]. A ZOTU table was then corrected for the 16S copy number based on the UNBIAS algorithm [46]. Phylogenetic affiliations were analyzed by the USEARCH SINTAX algorithm using a confidence threshold of 0.8 [45, 47]. Both OTUs and ZOTUs were compared against three databases: Ribosomal Database Project (RDP) 16S rRNA gene training set version 16 [48], SILVA database for ARB for small subunit ribosomal RNAs version 138 for 18S rRNA, and Unite database for ITS1 [49]. Moreover, OTUs and ZOTUs detected in control samples were used to identify cross-talk errors among the analyzed tardigrades samples, while the UNCROSS2 algorithm was used to remove OTUs and ZOTUs detected in control samples [47]. In addition, reads were normalized by OTUTAB_RARE algorithm [50] to compare sample diversities.

### Statistical methods

We investigated differences in bacteria, fungi and other micro-eukaryote communities using redundancy analyses (RDA) based on Hellinger distances. Alpha diversity was summarized using the Shannon diversity indices (base *e*) and the number of OTUs or ZOTUs. Variation in these indices among cryoconite, fully fed tardigrades and starved tardigrades was investigated using Generalized Linear Models (GLMs) assuming a Gaussian (Shannon index) or a Poisson (number of OTUs oZOTUs) distribution and a log link. The Poisson models showed under-dispersion (dispersion parameter ≤ 0.11), but conservatively we did not correct for it. Significance of the Gaussian models was checked with a permutation approach to account for potential slight deviations from model assumptions. Since results from randomization always confirmed those of the parametric model, only the latter were reported.

We also conducted an indicator species analysis to identify taxa associated with cryoconite, fully fed tardigrades and starved tardigrades. This analysis was done using the Indicator Value method [51], as implemented in the multipatt function of the INDICSPECIES package of R. All analyses were run in R 3.6.1 [52]. R script is attached in S1 File.

## Results

**Fig 2:**
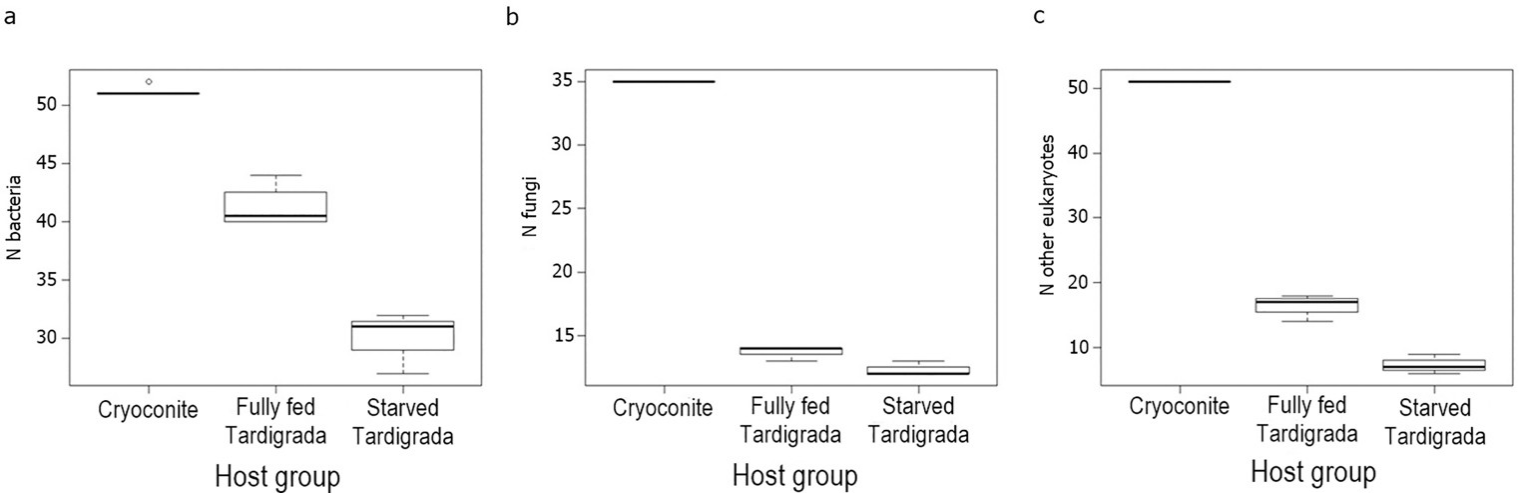
Boxplots of number of bacteria (ZOTU), fungi and microeukaryotes (OTU) taxa in cryoconite (six replicates = 6 x 1 millilitre), fully fed Tardigrada (four replicates = 4 x 10 specimens) and starved Tardigrada (three replicates = 3 x 10 specimens).

Overall, sequencing produced 48,810,016 reads. After quality filtering (low quality sequences as well as sequences detected in control samples), prokaryotic, fungal and other eukaryotic samples yielded 6,139,290, 5,162,132 and 24,311,639 reads, respectively. Sequences representing tardigrades were removed, therefore the final eukaryotic library consisted of 8,600,468 reads. The average number of reads from cryoconite samples was: 1,208,083 (V9 18S rDNA), 798,044 (V4 16S rDNA) and 727,194 (ITS1). From all tardigrades it was 20,556, 193,004, and 11,596 for microeukaryotes, bacteria and fungi, respectively. OTUs clustering across samples produced 133 unique OTUs for V9 18S rDNA and 51 unique OTUs for ITS1. Among V4, 16S rDNA sequences, 849 unique ZOTUs were clustered. Among them, 50 eukaryotic, 65 prokaryotic, and 34 fungal OTUs or ZOTUs were considered as “common” (Fig 2, S1-S6 Figs in S1 File). Both OTUs and ZOTUs defined as “common” had an abundance higher than 1% in at least one sample; the remaining were defined as “others”. The sequences are deposited in GenBank under: MW282178-MW282322 for bacteria, MW306127-MW306189 for fungi, MW306044-MW306126 for other microeukaryotes.

**Fig 3:**
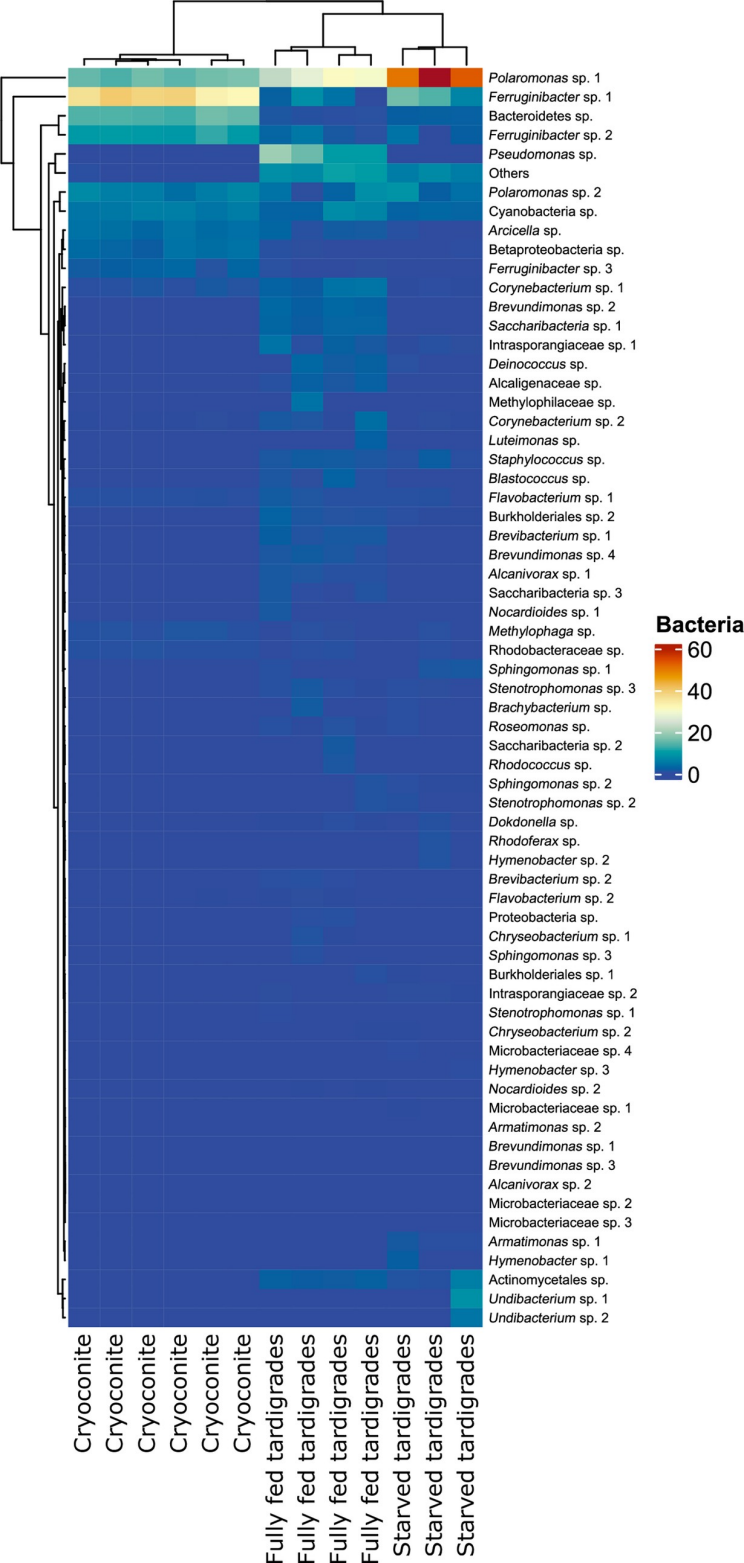
Heatmap of bacteria in different host groups (cryoconite, fully fed Tardigrada, starved Tardigrada). Darker colours represent higher abundance. Dendrograms highlight clustering of sites and taxa.

**Fig 4:**
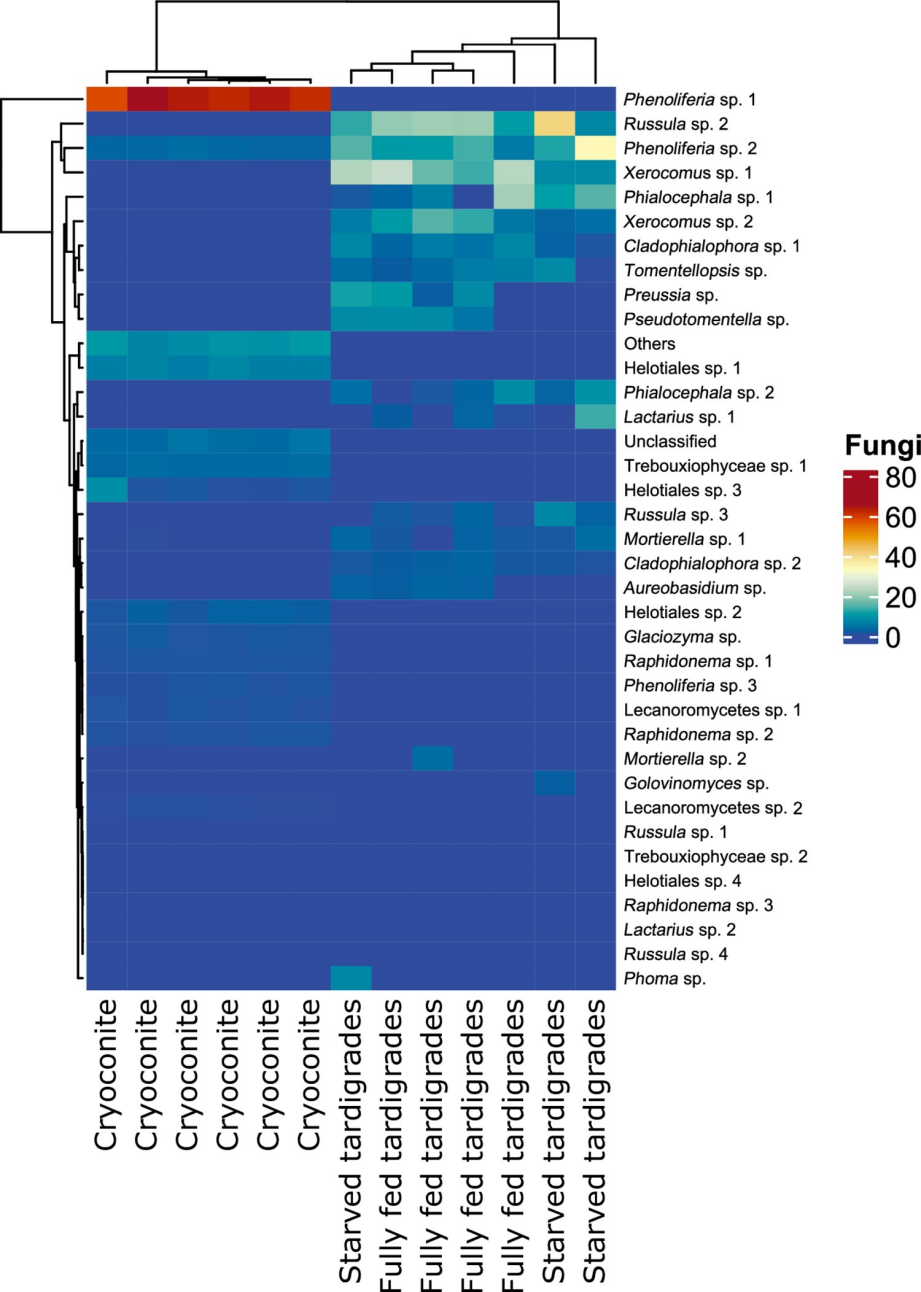
Heatmap of fungi in different host groups (cryoconite, fully fed Tardigrada, starved Tardigrada). Darker colours represent higher abundance. Dendrograms highlight clustering of sites and taxa.

**Fig 5:**
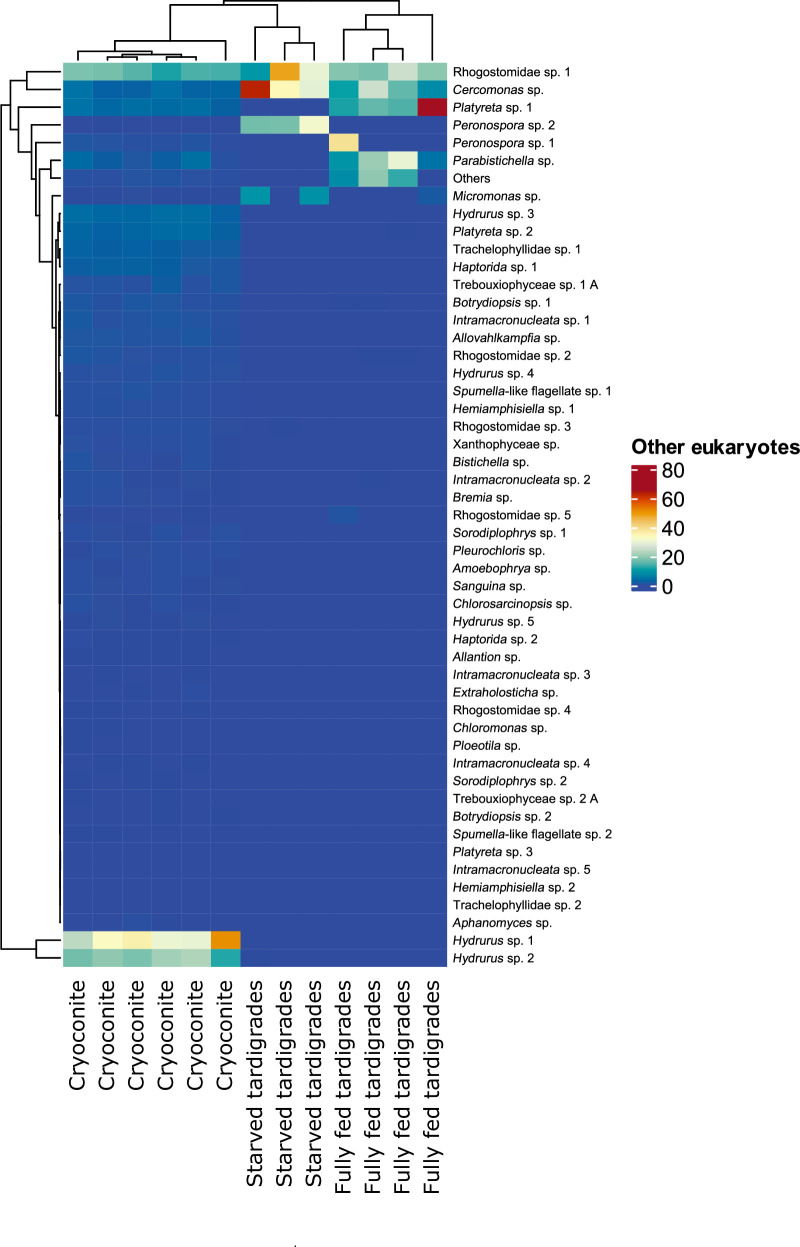
Heatmap of microeukaryotes in different host groups (cryoconite, fully fed Tardigrada, starved Tardigrada). Darker colours represent higher abundance. Dendrograms highlight clustering of sites and taxa.

**Fig 6:**
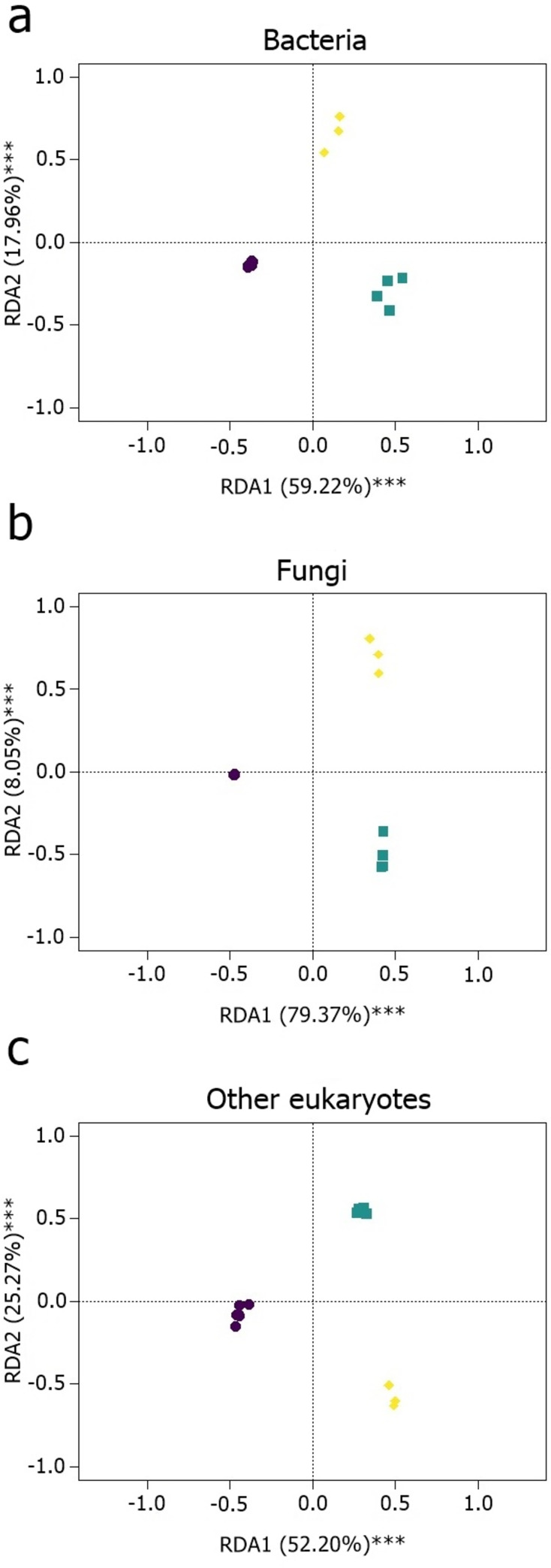
Distance biplots of RDAs for a) bacteria, b) fungi, c) other microeukaryotes. Violet circles = cryoconite, green squares = fully fed Tardigrada, yellow diamonds = starved Tardigrada.

**Fig 7:**
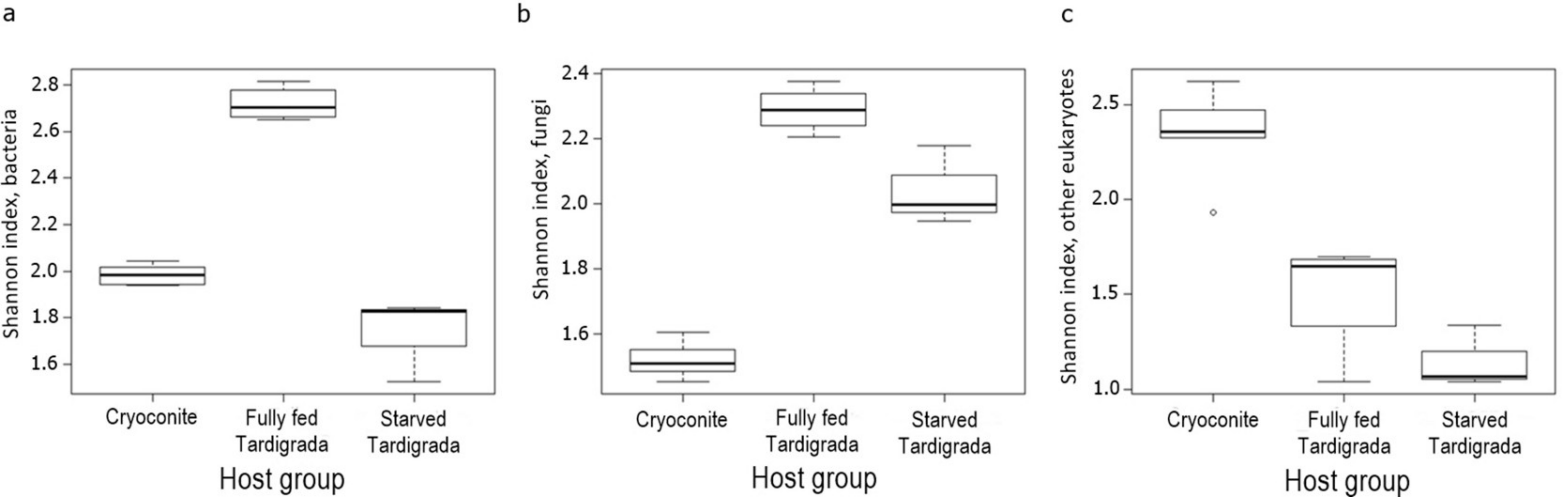
Boxplots of Shannon diversity indices of bacteria, fungi and other microeukaryotes in cryoconite, fully fed Tardigrada and starved Tardigrada.

The heatmap which visualizes the differences in the taxon (OTUs and ZOTUs) abundances among host groups (cryoconite, fully fed tardigrades and starved tardigrades), showed clustering for each microbial groups. The taxon composition differs between host groups for both bacteria and microeukaryotes, while for fungi differs between tardigrades and cryoconite, with no difference between fully fed and starved tardigrades (Figs 3–5). Separate Redundancy Analysis (RDAs) for bacteria, fungi and other eukaryotes showed significant differences among cryoconite, fully fed tardigrades and starved tardigrades for all taxonomic groups (Fig 6, Table 1). Relative numbers of taxa (OTUs and ZOTUs) as well as Shannon diversity indices showed significant differences between host groups for bacteria, fungi and other microeukaryotes (Figs 2 and 7). Nineteen ZOTUs of bacteria, 4 OTUs of fungi, and 8 OTUs of other microeukaryotes were common for cryoconite and fully fed tardigrades. While, indicator species analyses showed that 11 bacterial ZOTUs, 3 fungal OTUs, and 7 microeukaryotic OTUs were significantly associated with cryoconite and fully fed tardigrades (S2, S4, S6 Figs and S2-S4 Tables in S1 File).

**Table 1:** Separate RDAs of bacteria, fungi and other microeukaryotes communities according to host group (cryoconite, fully fed tardigrades, starved tardigrades).

Taxon	Explained Variance	Residual Variance	Adjusted-R2	F	df	P
Bacteria	0.211	0.050	0.772	21.287	2,10	0.001
Other eukaryotes	0.338	0.078	0.775	21.630	2,10	0.001
Fungi	0.470	0.055	0.874	42.717	2,10	0.001

### Bacteria

Overall, we found 65 ZOTUs of bacteria that occurred in both cryoconite and tardigrades (Figs 2 and 3, S1, S2 Figs in S1 File). ZOTUs abundances among host groups (cryoconite, fully fed tardigrades and starved tardigrades) differed (Fig 3). Among them 2 and 5 ZOTUs were unique for fully fed and starved tardigrades, while no unique ZOTUs were found for cryoconite. Twenty eight ZOTUs were common for all three groups (S2 Fig in S1 File). The highest relative abundant ZOTU shared between cryoconite, fully fed tardigrades and starved tardigrades was *Polaromonas* sp. 1 (14%-16%, 22%-31%, 50%-51% for each group, respectively). The most abundant ZOTU in cryoconite was *Ferruginibacter* sp. 1 with 31%-39% occurrence. Besides *Polaromonas* sp. 1, the second most abundant ZOTU in a fed tardigrade was *Pseudomonas* sp. (9%-19%) while in starved tardigrades it was *Ferruginibacter* sp. 1 (6%-16%) (see Fig 3, S1 Fig in S1 File). Indicator species analyses showed that two ZOTUs were significantly associated with cryoconite only, one with fully fed tardigrades, three with starved tardigrades, eleven with cryoconite and fully fed tardigrades, and five for both fully fed and starved tardigrades (see S2 Table in S1 File).

The relative richness of ZOTUs was highest in cryoconite, significantly lower in fully fed tardigrades and lowest in starved tardigrades (Fig 2). The Shannon diversity index showed significant differences between cryoconite fully fed and starved tardigrades. The highest Shannon index was detected in fully fed tardigrades, then in cryoconite and the lowest in starved tardigrades (Fig 7). Post-hoc tests (Tukey method) showed significant differences in alpha diversity between i) cryoconite and starved tardigrades, and ii) fully fed tardigrades and starved tardigrades (|t| ≥ 2.430, P ≤ 0.039), but the number of bacterial taxa was not significantly different for cryoconite and fully fed tardigrades (z = -2.232, P = 0.065).

### Fungi

Overall, we found 36 OTUs of fungi in cryoconite and tardigrades (Figs 2 and 4, S2 Fig in S1 File). OTUs abundances among host groups (cryoconite, fully fed tardigrades and starved tardigrades) differed (Fig 4). Among them 19, 0 and 2 OTUs were unique for cryoconite, fully fed and starved tardigrades, respectively. Twelve OTUs were common for all three groups (S4 Fig in S1 File). The highest relative abundant OTU of fungi in cryoconite was *Phenoliferia* sp. 1 with 62%-64% occurrence. In fully fed tardigrades, the most abundant OTUs were *Russula* sp. 2 (18%-20%), *Xerocomus* sp. 1 (13%-24.5%), and *Phenoliferia* sp. 2 (10–17%). In starving tardigrades the most relatively abundant were *Phialocephala* sp. 1 (11%-21%), *Russula* sp. 2 (7%-40%), *Phenoliferia* sp. 2 (5%-33%) (see S3 Fig in S1 File). Results of indicator species analyses selected 22 OTUs. Nineteen OTUs were associated with cryoconite only, while three OTUs were significantly associated to fully fed tardigrades only. We did not detect any common fungi OTUs shared between fully fed and starved tardigrades (S3 Table in S1 File). The common fungi for cryoconite and fully fed tardigrades, according to indicator species analyses, were *Aureobasidium* sp., *Preusia* sp., and *Pseudotomentella* sp.

The relative richness of fungal OTUs was the highest in cryoconite, significantly lower in fully fed tardigrades and the lowest in starving tardigrades (Fig 2). The number of fungal taxa was significantly different between cryoconite and tardigrades, both starved and fully fed (|t| ≥ 2.430, P ≤ 0.039). However, no significant difference between starved and fully fed tardigrades (t = -0.511, P = 0.863) was found (Table 2). The Shannon diversity index showed significant differences between cryoconite and tardigrades (Fig 7, Table 3). The highest values of the Shannon index were found in fully fed tardigrades, then in starved tardigrades and the lowest in cryoconite.

**Table 2:** Alpha diversity of bacteria, fungi and other microeukaryotes communities according to host group. Poisson models of the number of bacteria, fungi and microeukaryotes taxa in cryoconite, fully fed and starved tardigrades.

Taxon	χ^2^	df	P
Bacteria	22.24	2	< 0.001
Other eukaryotes	175.80	2	< 0.001
Fungi	67.60	2	< 0.001

**Table 3:** Shannon diversity indices between bacteria, fungi and other microeukaryotes, and host group (cryoconite, fully fed tardigrades, starved tardigrades). Gaussian models of Shannon diversity indices of bacteria, fungi and microeukaryotes communities in host groups. The significance of Gaussian models was checked with a permutation approach to account for potential slight deviations from model assumptions.

Taxon	F	df	P
Bacteria	111.330	2,10	< 0.001
Other eukaryotes	27.511	2,10	< 0.001
Fungi	129.200	2,10	< 0.001

### Other microeukaryotes

Overall, 54 eukaryotic taxa were detected using 18S rDNA marker (Figs 2 and 5, S5 Fig in S1 File). OTUs abundances among host groups (cryoconite, fully fed tardigrades and starved tardigrades) differed (Fig 5). Most of them were heterotrophic protists, however algae (*Chloromonas* sp., *Sanguina* sp.) were also detected. Among them 29 OTUs were unique for cryoconite, while no unique OTU was found in fully fed and starved tardigrades. Twelve OTUs were common for all three groups (S6 Fig in S1 File). The relative abundance of particular OTUs indicated that the highest abundant OTUs in cryoconite, fully fed, and starved tardigrades, were classified as protists: *Rhogostomidae* sp. 1, *Cercomonas* sp. (mostly abundant in starved tardigrades) and *Platyreta* sp. 1 occurred in cryoconite and starved tardigrades. The most frequent OTU for each group was *Rhogostomidae* sp. 1 (11%-18% in cryoconite, 17%-25% in fully fed tardigrades and 9%-48% in starved tardigrades). The second most common taxon was *Cercomonas* sp. with increasing frequency from cryoconite (2%-4%), through the fully fed tardigrades (8%-25%), reaching the highest frequency in starved tardigrades (34%-63%), see S3 Fig in S1 File.

Indicator species analysis selected 43 OTUs, 34 were associated with cryoconite and nine with cryoconite and fully fed tardigrades, comprising protists and algae. Only one OTU was common for cryoconite and starved tardigrades (*Peronospora* sp. 2) (see S4 Table in S1 File). The highest relative number of eukaryotic taxa was found in cryoconite, then in fully fed tardigrades and last in starved tardigrades (Fig 2). The Shannon diversity index showed the same pattern (Fig 7). Post-hoc tests (Tukey method) showed significant differences in alpha diversity between i) cryoconite and starved tardigrades, and ii) cryoconite and fully fed tardigrades (|t_10_| ≥ 2.430, P ≤ 0.039), but the number of eukaryotic taxa was not significantly different for fully fed and starved tardigrades (t_10_ = -1.901, P = 0.188).

## Discussion

Our work presents the first analysis of the microbiota of animals inhabiting glacial environments by comparing three host groups (cryoconite–the substratum and potential food source, fully fed and starved animals) and three genetic markers (16S rDNA for bacteria, ITS1 for fungi, and 18S rDNA for other microeukaryotes). Although commonly used in studies on microbiota, our analysis showed that molecular tools might be also useful in searching for putative food in microscopic animals extracted from environmental samples. The significant differences we found in relative abundance and diversity between cryoconite, fully fed and starved tardigrades as well as species indicator analysis suggest that, although microscopic invertebrates inhabit and feed on cryoconite, they host their own specific microbial communities (Figs 3, 4, 5 and S2, S4, S6 Figs, S2-S4 Tables in S1 File). In particular, the significant differences in abundance and diversity of all three microbial groups between fully fed and starved tardigrades indicate that the microbiota of microinvertebrates changes during induced stress (i.e., lack of food). Yet even starved tardigrades host diverse microbial communities. The finding of potential bacterial or fungal food in tardigrades such as *Flavobacterium* sp. or *Preussia* sp., suggests that, even though the psychrophilic tardigrade *C*. *klebelsbergi* is considered herbivorous (according to buccal tube morphology [24]), its diet may be more diverse and include other food sources.

### Bacteria

Studies of the bacterial microbiota of glacier invertebrates are rare [8–10]; regrettably, the same is true for representatives of the phylum Tardigrada, one of the most cosmopolitan and ubiquitous invertebrates [29, 53, 54]. Investigations of limnoterrestrial tardigrade microbiomes showed they were species-specific, but still moderated by the environment [29]. In addition, in our study, the number of ZOTUs and the Shannon diversity index of bacteria taxa was similar to those reported in previous studies [29]. The abundance and diversity of ZOTUs differs between host groups (Figs 2 and 3), which indicates each group is characterised by its own microbiome.

ZOTUs of *Flavobacterium* and *Ferruginibacter* were among the most abundant in fully fed tardigrades, consistent with the results of Vecchi et al. [29] and Murakami et al. [9]. However, their absence in starved tardigrades suggests that microbiome bacteria may change during starvation, as occurs in animals [55, 56] (Fig 3). In contrast, we did not find sequences belonging to *Rickettsiales*, which were indicated as potential symbionts of tardigrades and plecopterans by Vecchi et al. [29] and Murakami et al. [9], respectively.

The relative abundance of *Polarmonas* sp. (a bacterial taxon typically found in glaciers and other cold environments [9, 10]), was very high in all three groups (cryoconite, fully fed and starved tardigrades). The ubiquitous ZOTUs of *Polaromonas* sp. in the three host group is consistent with its dormancy-inducing capacity, which confers capability to survive under harsh conditions, and its metabolic versatility [57]. These traits might explain the high relative abundance of *Polaromonas* ZOTU in starved tardigrades, and in other glacier species [9, 10]. Interestingly, the *Polaromonas* phylotypes found in plecopterans were rarely detected in the glacier surface habitat by Murakami et al. [9], while we found the same *Polaromonas* ZOTU in cryoconite and animals. Previous studies inferred a symbiotic association between *Polaromonas* species and snow algae [58, 59]. Therefore, Murakami et al. [9] stated that such symbioses between glacier-indigenous microbes and glacier eukaryotes, including invertebrates, may be a common phenomenon in glacier ecosystems.

According to the species indicator analysis, only *Flavobacterium* sp. 2, among the most abundant taxa, was statistically typical of both cryoconite and fully fed tardigrades, while ZOTUs of *Polaromonas* sp. and *Ferruginibacter* sp. were neither specific to cryoconite or tardigrades (both fully fed and starved). *Flavobacterium* sp. is a common taxon in soil and aquatic habitats, thus it might be eaten by tardigrades along with other environmental bacteria found in cryoconite (S2 Table in S1 File). Among bacteria taxa in cryoconite, 11 were common in both tardigrades and cryoconite, so food selection cannot be excluded. In agreement with the results of our analyses of the relative number and the Shannon diversity index of bacteria taxa, previous investigations on limnoterrestrial tardigrade microbiomes showed they were species-specific, but still moderated by the environment [29].

Five bacteria ZOTUs were common for fully fed and starved tardigrades which suggests they are part of their microbiome (S2 Fig, S2 Table in S1 File). Among them, *Intrasporangiaceae* sp. 1, *Staphylococcus* sp., *Stenotrophomonas* sp. 3, are commensals of other animals, while the roles of *Actinomycetales* sp. and *Burkholderiales* sp. 2 are enigmatic and complex [60, 61]. Coagulase-negative *Staphylococcus* spp. has been associated with mammals and birds [62], and a *Staphylococcus* strain, isolated in Antarctica, has been recently described as a new species potentially adapted to extreme environments [63]. It is therefore not surprising to find a representative of this genus in cryoconite and associated with tardigrades. The order *Actinomycetales*, which also includes the family *Intrasporangiaceae*, comprises physiologically diverse groups of bacteria, mainly found in soils, but also associated with eukaryotic hosts in diverse niches, such as the exoskeleton of some tropical ants, the lungs and skin of mammals and insects, and the roots and inner tissues of plants [64]. The genus *Burkholderia*, which is in the order *Burkholderiales*, contains metabolically versatile bacteria, also known as insect symbionts. Interestingly, in contrast to many other insect symbioses, the known associations with *Burkholderia* are characterized by environmental symbiont acquisition [65].

With the exception of *Polaromonas* ZOTU, the relative abundance of dominant bacterial taxa in *C*. *klebelsbergi* differed from those found by Murakami et al. [8] in glacier ice worms. This might be explained not only by the obvious differences between the model organisms (Tardigrada vs Oligochaeta), but also by the different types of glacier environments where these organisms live. Ice worms inhabit the englacial zone/firn above the equilibrium line altitude on glaciers [66] while *C*. *klebelsbergi* is mostly linked to cryoconite [21]. Murakami et al. [8] suggested that the intestinal tract of the ice worm appears to provide a unique habitat, which is potentially rich of nutrients, necessary for microbes in glacial ice ecosystems with limited resources, and shape a worm-associated bacterial microbiota that is distinct from glacial habitats. Similar conclusions may not hold for tardigrades, as Forni cryoconite is rather nutrient-rich. Further studies are therefore necessary to assess the microbiota associated with glacier invertebrates, what kind of food invertebrates utilize on glaciers and how bacteria shape invertebrate assemblages and *vice versa*.

### Fungi

Fungi are present in all ecological niches of glacial ecosystems [67]. Although fungi have been described by the first explorers of cryoconite [68], knowledge on this important taxon on ice and in cryoconite is fragmentary. Previous studies focused mostly on typical antagonistic relations [33–69], without any consideration of fungi as a food or symbionts for tardigrades. The abundance and diversity of OTUs differs between tardigrades and cryoconite (Fig 4), which indicates each host group is characterized by its own microfungal community. However, opposite to bacteria, we did not detected clear differences between fully fed and starved tardigrades.

Some of the taxa we found on Forni are typical cold-dwelling fungi, however many are not, and they are most probably allochthonous, transported from valley, mountain slopes, or by tourists. The taxon with the largest relative abundance in cryoconite was *Phenoliferia* sp. 1, a typical cryophilic fungi related to other cold adapted yeasts that has been detected also on other glaciers [70]. However, this species was not common in tardigrades, both fully fed and starved. We also found unexpectedly high relative abundances of *Russula* sp. 2 and *Xerocomus* sp. 1 (two fungal genera common in forests and with specimens of large size) in fully fed tardigrades (Fig 5, S4 Fig in S1 File), which suggests that these fungi (or, most probably, their fragments or spores) were passively delivered to the glacier, and eaten by tardigrades just before their collection. The relative high abundance of *Phialocephala* sp., an ectoparasite of plants [71], in hungry tardigrades was unexpected. Possibly the spores of this taxon could survive in tardigrades during the process of starvation, thus showing a high relative abundance of this taxon.

Indicator species analysis showed that only three fungal taxa, *Aureobasidium* sp., *Preussia* sp., and *Pseudotomentella*, were typical of both cryoconite (a potential source of food) and fully fed tardigrades (S3 Table in S1 File). *Aureobasidium* is a black yeast parasitic on plants and apparently was delivered on ice with leaves and spruce needles (observed during fieldworks). *Preussia* sp. was previously detected in cryoconite in Svalbard and Greenland [70–72], it is widespread in soils and may be part of tardigrade diet. *Pseudotomentella* sp. is an ectomycorrhizae fungus [73], whose presence on ice might be related to allochtonous organic material delivered to the glacier, as for *Aureobasidium* sp.

Generally, the presence of plant pathogens on ice is not surprising because many fungal taxa are recognized as endophytes, and plant pathogens are known to occur in supraglacial environments characterized by a high abundance of ice algae [72]. The glacier surface is a repository of various biogenic materials both windblown and delivered directly by animals [21–74], probably including humans.

### Other eukaryotes

Sequencing of the 18S rDNA marker in a cryoconite environment is crucial for the identification of many invisible eukaryotic taxa [75]. These fragile organisms, such as soft bodied plathelyminthes or amoebas, are mostly destroyed and invisible with a microscope after preservation [76], or destroyed during refreezing and observation on a microscope slide (E. Poniecka pers. comm.).

The abundance and diversity of OTUs differs between host groups (Fig 5), which indicates each group is characterized by its own microeukaryotic community. The role of microeukaryotes inside microinvertebrate bodies remains uncertain. Some might be parasites, commensals, food or just randomly consumed elements [77]. Studies on microeukaryotes in tardigrades are related to fungi and a few protozoans, which are mostly parasitic [33]. Here, for the first time, we showed the composition of microeukaryotes from cryoconite and tardigrades using an NGS approach and revealed the potential links of tardigrades with putative parasites and food originating from cryoconite. In our study, only seven eukaryotes were common in both cryoconite and tardigrades. *Trebouxiophyceae* sp. 1A (Chlorophyta) and *Xanthophyceae* sp. (Ochrophyta), so called yellow-green algae, are most probably food of tardigrades, although phylogenetically distant, they are similar to algae eaten by tardigrades both in laboratory cultures [78], and in natural environments [79]. Protists (*Parabistichella* sp., *Platyreta* sp. 1, sp. 2, *Rhogostomidae* sp. 2, and *Intramacronucleata* sp. 3) were probably eaten from cryoconite by chance, because they are not typical food for tardigrades (at least not known until now). *Platyreta* sp. is an amoeba known as parasite of algae species but also nematodes [80]. On glaciers, it can live as a predator or parasite of ice algae, or parasite of tardigrades, but such a relationship is highly speculative without additional testing. Interestingly, we detected also reads of the snow algae *Sanguina* sp. [81]. Although, their relative abundance in cryoconite and fully fed tardigrades was very low (ca. 0.1%-0.4% in cryoconite and 0.03%-0.05% in fully fed tardigrades), we cannot exclude that this taxon is food for tardigrades on glaciers. Visual observations of intestines of tardigrades from cryoconite in Arctic regions show they are often reddish, indicative of the consumption of pigmented Chlorophyta-like snow *Sanguina* sp. or ice algae (Streptophyta). Moreover, cryoconite granules, which are “feeders” for invertebrates are organic matter rich and contain many different algae species [17, 82]. The last two eukaryotes common in both cryoconite and tardigrades were *Rhogostoma* and *Cercozoa*, which were found also on glaciers in McMurdo Dry Valleys [75]. However, their role in the tardigrade microbiome remains enigmatic.

## Conclusion

This study presents novel analyses of cryoconite and tardigrades using three genetic markers for bacteria, fungi, and other microeukaryotes. Both relative abundance and species indicator analysis showed strong links between cryoconite and tardigrades indicating potential symbionts and food for these animals. The cryoconite not only provided potential food for tardigrades including bacteria and fungi, but also algae, the more typical food for these animals. Tardigrades are common and abundant grazers on glaciers and are an important biotic component of the ecosystem. In order to elucidate and estimate top-down control and secondary production in glacial ecosystems, knowledge on their diet and feeding modes is highly needed. Unfortunately, our experimental design did not allow us to evaluate whether the glacial tardigrade diet reflects food availability or food selectivity. The fact that some bacterial taxa were typical of both fully fed and starved tardigrades suggests that they are symbionts for these animals. The diverse microbial assemblages associated with tardigrades imply that microscopic metazoans contribute to cryoconite microbiome biodiversity, and their presence can enrich the microbiome of glaciers.

## Supporting information

S1 File S1-S6 Figs, S1-S4 Tables, R script. (PDF)
