# Supplementary material for: Trophic and symbiotic links between obligate-glacier water bears (Tardigrada) and cryoconite microorganisms
Source: PLoS One. 2022 Jan 12;17(1):e0262039. doi: 10.1371/journal.pone.0262039 (PMC8754347; doi:10.1371/journal.pone.0262039)
Supplement: S1 File — (PDF) [file pone.0262039.s001.pdf]

The supplementary materials

Krzysztof Zawierucha, Artur Trzebny, Jakub Buda, Elizabeth Bagshaw, Andrea Franzetti, Miroslawa Dabert, Roberto Ambrosini. ***Trophic and symbiotic links between obligate-glacier water bears (Tardigrada) and cryoconite microorganisms.***

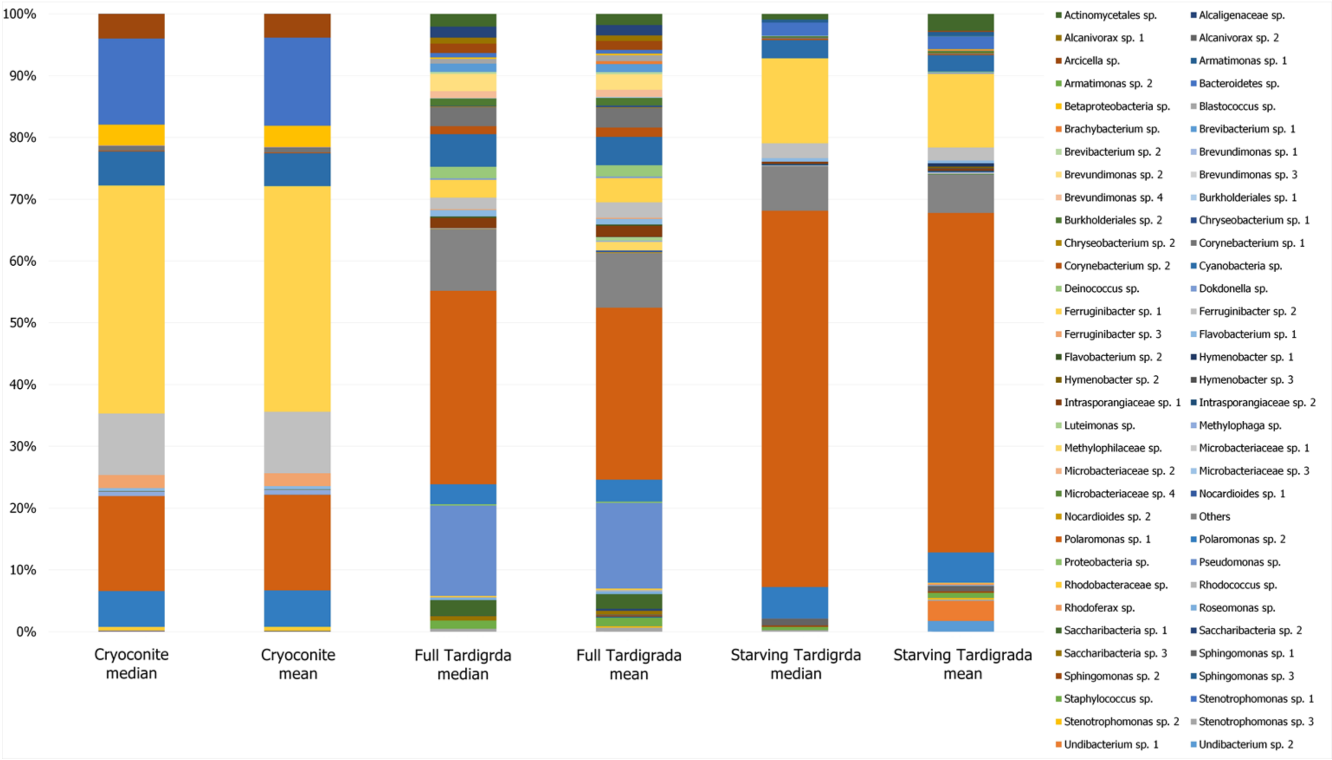

Supplementary figure 1. Relative frequency of bacteria in the host groups (cryoconite, fully fed Tardigrada, starved Tardigrada).

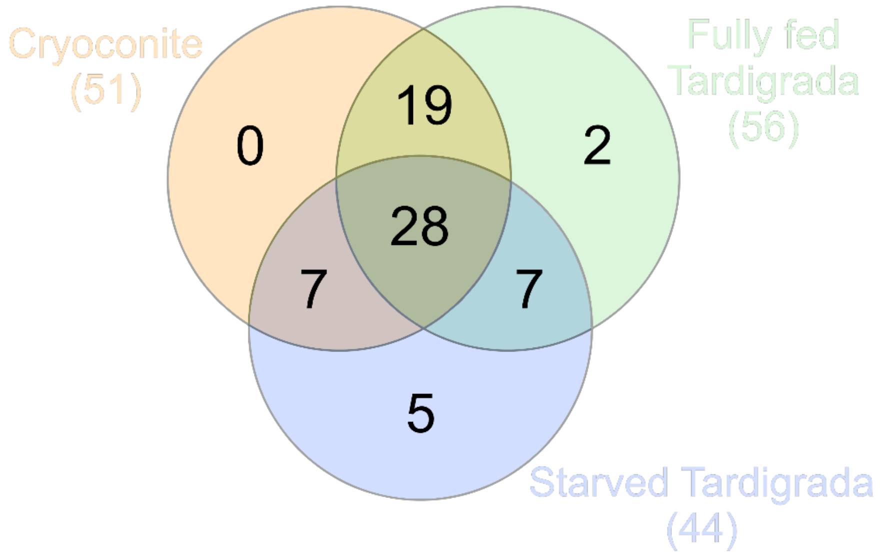

Supplementary figure 2. Venn diagrams representing number of common and unique bacterial ZOTUs for cryoconite, fully fed tardigrades and starved tardigrades.

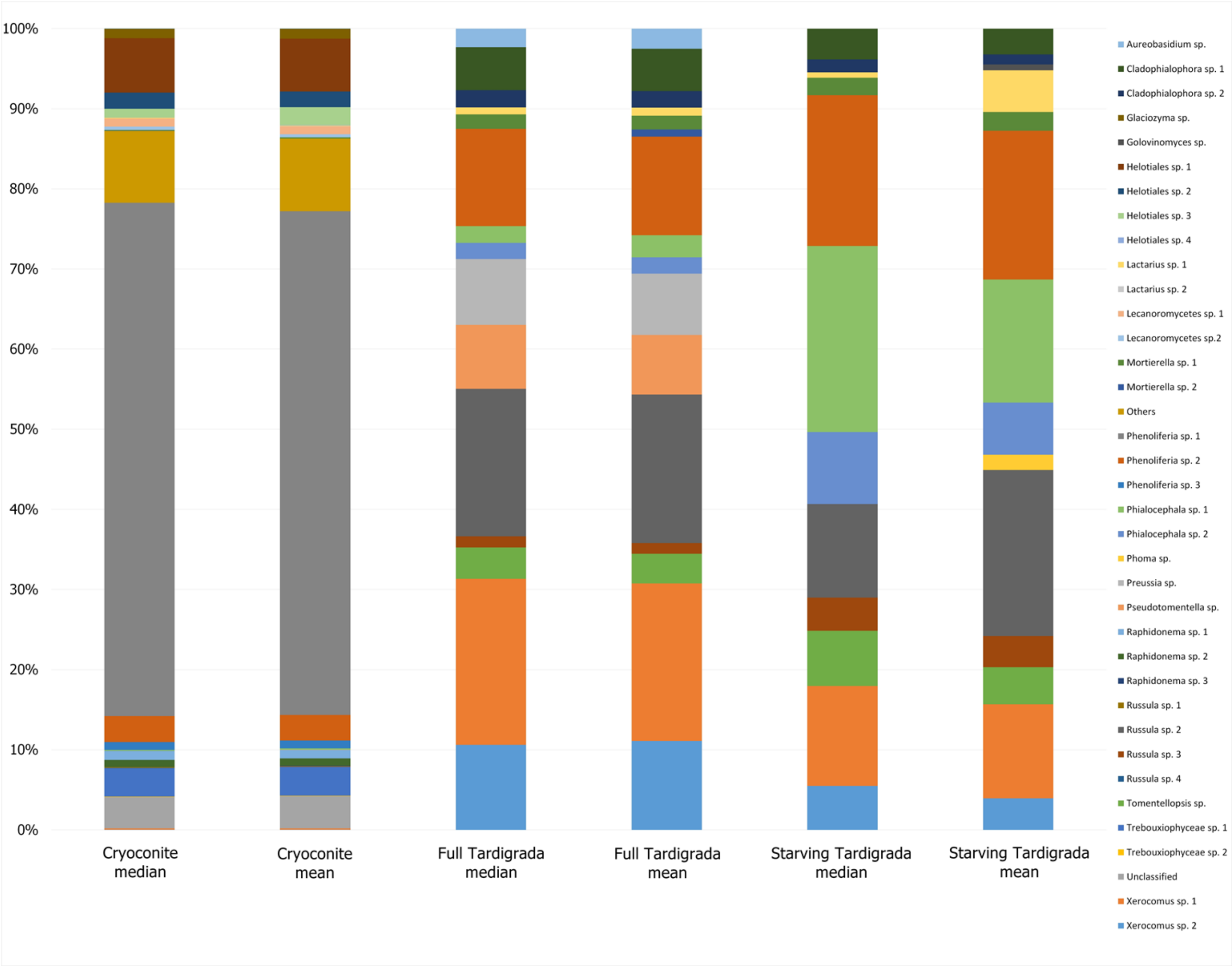

Supplementary figure 3. Relative frequency of fungi in the different host groups (cryoconite, fully fed Tardigrada, starved Tardigrada).

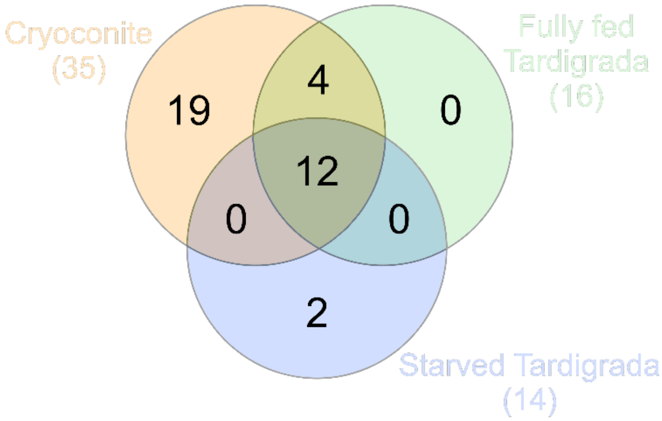

Supplementary figure 4. Venn diagrams representing number of common and unique fungal OTUs for cryoconite, fully fed tardigrades and starved tardigrades.

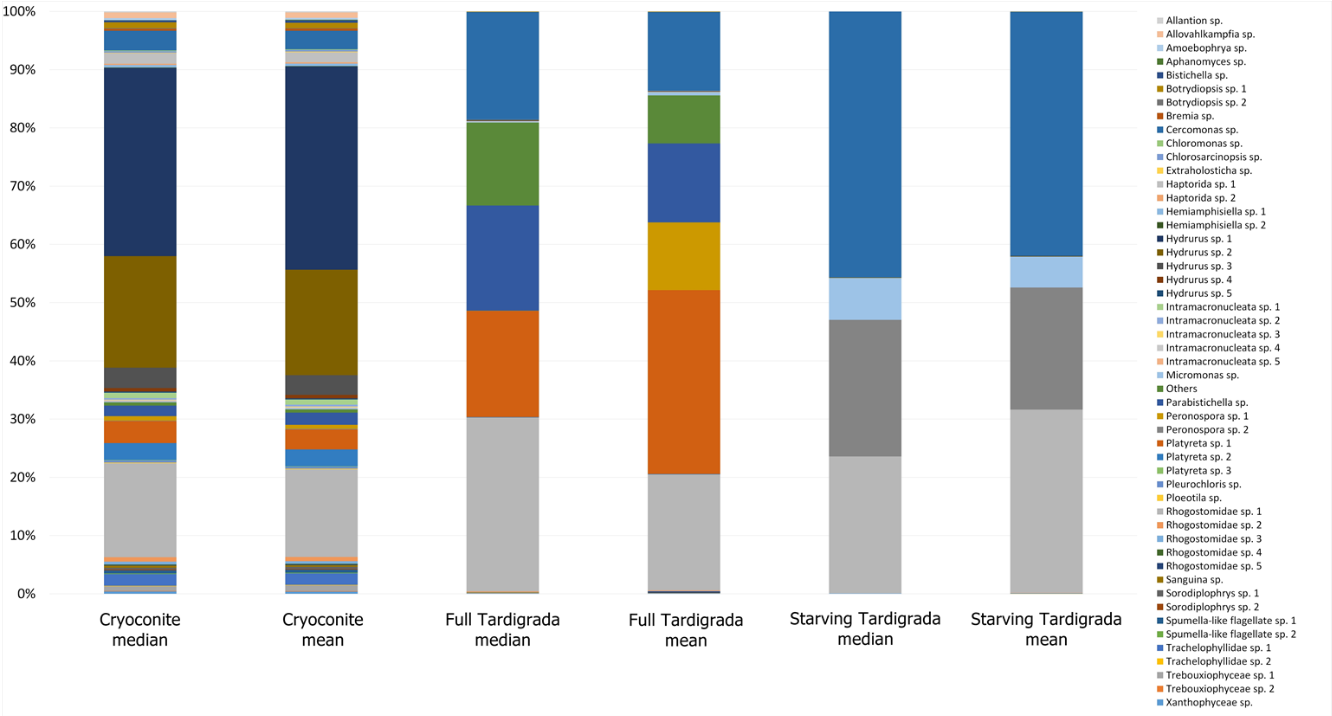

Supplementary figure 5. Relative frequency of microeukaryotes in the different host groups (cryoconite, fully fed Tardigrada, starved Tardigrada).

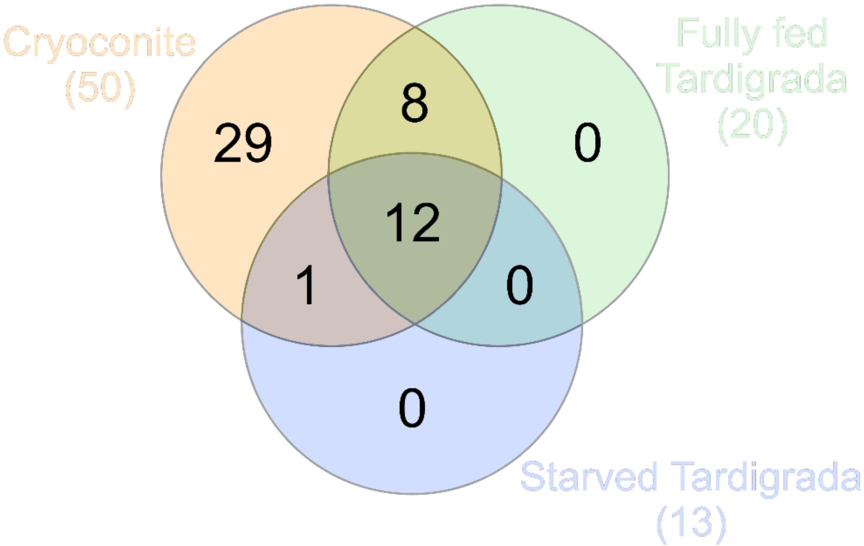

Supplementary figure 6. Venn diagrams representing number of common and unique microeukaryote OTUs for cryoconite, fully fed tardigrades and starved tardigrades.

Supplementary Table 1. All PCR primers for NGS sequencing used in this study.

Target	Primer	Sequence	Reference
V9 18 rDNA	1391F	GTACACACCGCCCGTC	Lane, 1991
	EukBr	TGATCCTTCTGCAGGTTCACCTAC	Medlin et al., 1988
V4 16 rDNA	V4F	CGATCAGCAGCCGCGGTAATA	Makowska et al. 2020
	V4R	ATGGACTACCAGGGTATCTAA	Therese et al., 1998
ITS1	ITS1	TCCGTAGGTGAACCTGCGG	White et al. 1990
	ITS2	GCTGCGTTCTTCATCGATGC	

Lane DJ (1991) 16S/23S sequencing. In:Nucleic Acid Technologies in Bacterial Systematic (eds Stackebrandt E, Goodfellow M). pp. 115–175, Wiley, NY.

Medlin LK, Elwood HJ, Stickel S, Sogin ML (1988) The characterization of enzymatically amplified eukaryotic 16S-like rRNA- coding regions. Gene, 71, 491–499.

Makowska N, Philips A, Dabert M, Nowis K, Trzebny A, Koczura R, et al. Metagenomic analysis of β - lactamase and carbapenemase genes in the wastewater resistome. Water Res. 2020;170.

Therese, K.L., Anand, A.R., Madhavan, H.N., 1998. Polymerase chain reaction in the diagnosis of uveitis. Br. J. Ophthalmol. 82 (9)

White TJ, Bruns T, Lee S, Taylor J (1990) Amplification and direct sequencing of fungal ribosomal RNA genes for phylogenetics. In: Innis DHGMA, Sninsky JJ, White TJ (Eds.), PCR Protocols: A Guide to Methods and Applications. Academic Press, New York, pp. 315–322.

Supplementary table 2-4.

Results from indicator species analyses Association function: IndVal.g, Significance level (alpha): 0.05.

Table 2. BACTERIA: Total number of species: 66. Selected number of species: 22. Number of species associated to 1 group: 6. Number of species associated to 2 groups: 16

Group Cryoconite #sps. 2

		A	B	stat	p.value
Ferruginibacter sp. 3		0.9146	1.0000	0.956	0.006
Betaproteobacteria sp.	0.9087	1.0000	0.953	0.006	

Group Full_Tardigrada #sps. 1

		A	B	stat	p.value
Roseomonas sp.	0.7598	1.0000	0.872	0.015	

Group Hungry_Tardigrada #sps. 3

		A	B	stat	p.value
Microbacteriaceae sp. 1	1.0000	1.0000	1.000	0.006	
Armatimonas sp. 1	0.9960	1.0000	0.998	0.006	
Intrasporangiaceae sp. 2	0.7833	1.0000	0.885	0.015	

Group Cryoconite+Full_Tardigrada #sps. 11

		A	B	stat	p.value
Alcaligenaceae sp.	1.0000	1.0000	1.000	0.006	
Alcanivorax sp. 1	1.0000	1.0000	1.000	0.006	
Blastococcus sp.	1.0000	1.0000	1.000	0.006	
Brevibacterium sp. 1		1.0000	1.0000	1.000	0.006
Brevibacterium sp. 2		1.0000	1.0000	1.000	0.006
Brevundimonas sp. 2		1.0000	1.0000	1.000	0.006
Brevundimonas sp. 4		1.0000	1.0000	1.000	0.006
Chryseobacterium sp. 1	1.0000	1.0000	1.000	0.006	
Saccharibacteria sp. 1	1.0000	1.0000	1.000	0.006	
Arcicella sp.		0.9666	1.0000	0.983	0.009
Flavobacterium sp. 2		1.0000	0.9000	0.949	0.019

Group Full_Tardigrada+Hungry_Tardigrada #sps. 5

		A	B	stat	p.value
Actinomycetales sp.	1	1	1	0.001	
Burkholderiales sp. 2		1	1	1	0.001
Intrasporangiaceae sp. 1	1	1	1	0.001	
Staphylococcus sp.	1	1	1	0.001	
Stenotrophomonas sp. 3	1	1	1	0.001	

Table 3. FUNGI: Total number of species: 37. Selected number of species: 22. Number of species associated to 1 group: 19. Number of species associated to 2 groups: 3.

Group	Cryoconite	#sps.	19		
		A	B	stat	p.value
Glaciozyma sp.	1	1	1	0.001	
Helotiales sp. 1	1	1	1	0.001	
Helotiales sp. 2	1	1	1	0.001	
Helotiales sp. 3	1	1	1	0.001	
Helotiales sp. 4	1	1	1	0.001	
Lactarius sp. 2	1	1	1	0.001	
Lecanoromycetes sp. 1	1	1	1	0.001	
Lecanoromycetes sp.2	1	1	1	0.001	
Phenoliferia sp. 1	1	1	1	0.001	
Phenoliferia sp. 3	1	1	1	0.001	
Raphidonema sp. 1	1	1	1	0.001	
Raphidonema sp. 2	1	1	1	0.001	
Raphidonema sp. 3	1	1	1	0.001	
Russula sp. 1		1	1	1	0.001
Russula sp. 4		1	1	1	0.001
Trebouxiophyceae sp. 1F	1	1	1	0.001	
Trebouxiophyceae sp. 2F	1	1	1	0.001	
Unclassified		1	1	1	0.001
Others F		1	1	1	0.001

Group	Cryoconite+Full_Tardigrada		#sps.	3	
		A	B	stat	p.value
Aureobasidium sp.	1	1	1	0.005	
Preussia sp.		1	1	1	0.005
Pseudotomentella sp.	1	1	1	0.005	

Table 4. **MICROEUKARYOTES**: Total number of species: 51. Selected number of species: 43. Number of species associated to 1 group: 34. Number of species associated to 2 groups: 9.

Group	Cryoconite	#sps.	34		
		A	B	stat	p.value
Allantion sp.		1.0000	1.0000	1.000	0.002
Allovahlkampfia sp.		1.0000	1.0000	1.000	0.002
Amoebophrya sp.	1.0000	1.0000	1.000	0.002	
Aphanomyces sp.	1.0000	1.0000	1.000	0.002	
Bistichella sp.		1.0000	1.0000	1.000	0.002
Botrydiopsis sp. 2	1.0000	1.0000	1.000	0.002	
Bremia sp.		1.0000	1.0000	1.000	0.002
Chloromonas sp.	1.0000	1.0000	1.000	0.002	
Chlorosarcinopsis sp.		1.0000	1.0000	1.000	0.002
Extraholosticha sp.	1.0000	1.0000	1.000	0.002	

Haptorida sp. 2		1.0000	1.0000	1.000	0.002
Hemiamphisiella sp. 1		1.0000	1.0000	1.000	0.002
Hemiamphisiella sp. 2		1.0000	1.0000	1.000	0.002
Hydrurus sp. 3		1.0000	1.0000	1.000	0.002
Hydrurus sp. 5		1.0000	1.0000	1.000	0.002
Intramacronucleata sp. 1	1.0000	1.0000	1.000	0.002	
Intramacronucleata sp. 4	1.0000	1.0000	1.000	0.002	
Intramacronucleata sp.	5	1.0000	1.0000	1.000	0.002
Platyreta sp. 3		1.0000	1.0000	1.000	0.002
Pleurochloris sp.	1.0000	1.0000	1.000	0.002	
Ploeotila sp.		1.0000	1.0000	1.000	0.002
Rhogostomidae sp. 4		1.0000	1.0000	1.000	0.002
Sorodiplophrys sp. 1		1.0000	1.0000	1.000	0.002
Sorodiplophrys sp. 2		1.0000	1.0000	1.000	0.002
Spumella-like flagellate sp. 1	1.0000	1.0000	1.000	0.002	
Spumella-like flagellate sp. 2	1.0000	1.0000	1.000	0.002	
Trachelophyllidae sp. 1		1.0000	1.0000	1.000	0.002
Trachelophyllidae sp. 2		1.0000	1.0000	1.000	0.002
Trebouxiophyceae sp. 2A	1.0000	1.0000	1.000	0.002	
Hydrurus sp. 1		0.9981	1.0000	0.999	0.008
Hydrurus sp. 2		0.9929	1.0000	0.996	0.027
Haptorida sp. 1		0.9745	1.0000	0.987	0.002
Hydrurus sp. 4		0.9467	1.0000	0.973	0.002
Botrydiopsis sp. 1	0.8722	1.0000	0.934	0.022	
Group Cryoconite+Full_Tardigrada			#sps.	8	
		A	B	stat	p.value
Parabistichella sp.	1.0000	1.0000	1.000	0.005	
Platyreta sp. 1		1.0000	1.0000	1.000	0.005
Platyreta sp. 2		1.0000	1.0000	1.000	0.005
Rhogostomidae sp. 2		0.9723	1.0000	0.986	0.016
Trebouxiophyceae sp. 1A	0.9700	1.0000	0.985	0.019	
Intramacronucleata sp. 3	0.9074	1.0000	0.953	0.007	
Xanthophyceae sp.	1.0000	0.9000	0.949	0.028	
Others A		1.0000	0.9000	0.949	0.035
Group Cryoconite+Hungry_Tardigrada				#sps.	1
		A	B	stat	p.value
Peronospora sp. 2	1	1	1	0.001	

Supplementary R script 1. Script for the analysis conducted within this study.

```
# preliminary operations
source("C:/Users/rober/OneDrive - Universitŕ degli Studi di Milano/Documenti/Documenti/Script statistica/Funzioni R/mie funzioni R.txt")

# import data
library(readxl)
library(BiodiversityR)
ZOTU <- data.frame(read_excel("C:/Users/rober/OneDrive - Universitŕ degli Studi di Milano/Documenti/Documenti/Dati/Ghiacciaio Forni/Tardigrades/ITS_16S_18S_no norm.xlsx", "TOTAL - ALL (without norm)"))

head(ZOTU)
dim(ZOTU)

Tax <- ZOTU[,1:3]
BacOLD <- ZOTU[,4:22]
rownames(BacOLD) <- Tax$X.OTU

names(BacOLD)
BacOLD <- BacOLD[,!grepl("mean", names(BacOLD))]
BacOLD <- BacOLD[,!grepl("median", names(BacOLD))]

dim(BacOLD)
head(BacOLD)
head(Tax)
table(Tax$Target)

# some descriptive statistics

BacOLD2 <- t(BacOLD)
BacOLD2[1:5,1:5]


Algae <- BacOLD2[,Tax$Target=="Algae"]
Bacteria <- BacOLD2[,Tax$Target=="Bacteria"]
Fungi <- BacOLD2[,Tax$Target=="Fungi"]


# change target taxon for RDA analyses
```

```
Bac <- Fungi

head(Bac)

heatmap(Bac, margins=c(8,8))
reset_par()

Env <- data.frame(Treat=rownames(Bac))
Env$Treat <- gsub('.{3}$', '', Env$Treat)
Env$NOTUs <- apply(I(BacOLD2>0), 1, sum)
boxplot(NOTUs ~ Treat, data = Env)


Env$NAlgae <- apply(I(Algae>0), 1, sum)
Env$NBacteria <- apply(I(Bacteria>0), 1, sum)
Env$NFungi <- apply(I(Fungi>0), 1, sum)

boxplot(NAlgae ~ Treat, data = Env)
boxplot(NBacteria ~ Treat, data = Env)
boxplot(NFungi ~ Treat, data = Env)


BacHelli <- disttransform(Bac, method = "hellinger")

# RDA
RDAB <- rda(BacHelli ~ Treat , data = Env)

RsquareAdj(RDAB)$adj.r.squared

over.B <- anova(RDAB, permutations = how(nperm =999))
over.B

#
margin.B <- anova(RDAB, by="margin", permutations = how(nperm =999))
margin.B

axes.B <- anova(RDAB, by="axis", permutations = how(nperm =999))
axes.B

POST <- multiconstrained(method="rda", BacHelli ~ Treat, data = Env, distance = "euclidean")
POST
```

```
# RDA plot

ooo <- summary(RDAB, scaling=2)
ooo$cont$importance[,1:3]

# adjusted proportion of variance explained

GR <- grep("RDA",colnames(ooo$cont$importance))
if(sjmisc::is_empty(GR)){
  APVE <- ooo$cont$importance[2,1:2]
} else {
  if(max(GR) >= 2){
    APVE                                                                    <-
ooo$cont$importance[2,1:2]*RsquareAdj(RDAB)$adj.r.squared/sum(ooo$cont$importance[2,1:2])
  }
  if(max(GR) == 1){
    APVE <- ooo$cont$importance[2,1:2]
    APVE[1] <- RsquareAdj(RDAB)$adj.r.squared
  }}



# asterisks
Ast1 <- c("", "")
if(!sjmisc::is_empty(GR)){
  if(max(GR) >= 1){
    if(axes.B[4]$`Pr(>F)`[1]<=0.05) Ast1[1] <- "*"
    if(axes.B[4]$`Pr(>F)`[1]<=0.01) Ast1[1] <- "**"
    if(axes.B[4]$`Pr(>F)`[1]<=0.001) Ast1[1] <- "***"
  }
  if(max(GR) >= 2){
    if(axes.B[4]$`Pr(>F)`[2]<=0.05) Ast1[2] <- "*"
    if(axes.B[4]$`Pr(>F)`[2]<=0.01) Ast1[2] <- "**"
    if(axes.B[4]$`Pr(>F)`[2]<=0.001) Ast1[2] <- "***"
  }}


AxLab <- paste(names(APVE), " (",
               format(round(APVE*100, 2), nsmall=2),
               #round(APVE*100, 2),
               "%) ", Ast1, sep="")
```

```
Plot <- ordiplot(RDAB, choices = c(1,2),  scaling=2)

# correlation between distances in the graph and real distances
DD <- distdisplayed(BacHelli, ordiplot=Plot, distx = "euclidean", plotit = TRUE, gam=TRUE,
                    method = "pearson")
rs <- round(DD$mantelanalysis$statistic, 3)
rs

Plot <- ordiplot(RDAB, choices = c(1,2),  scaling=2, type='n',
                #display="sites",
                main = "Fungi",
                las=1,
                xlim=c(-1,1), ylim=c(-1,1),
                xlab=AxLab[1], ylab=AxLab[2],
                cex.axis=1.2, cex.lab=1.2)

# add surface of environemntal variables
#with(EnvFS, ordisurf(RDAB, Year, scaling=1, add = TRUE, col = "green4"))
#with(EnvFS, ordisurf(RDAB, condition, scaling = 1, add = TRUE, col = "red"))

# colors according to glacier

Symb <- rep(19, nrow(Env)) # Cryoconite = circle
Symb[Env$Treat=="Full_Tardigrada"] <- 15 # square
Symb[Env$Treat=="Hungry_Tardigrada"] <- 18 # diamond

library(viridis)
Mycol <- viridis(3)

Col <- rep(Mycol[1], nrow(Env))
Col[Env$Treat=="Full_Tardigrada"] <- Mycol[2]
Col[Env$Treat=="Hungry_Tardigrada"] <- Mycol[3]


points(RDAB, "sites", choices = c(1,2), scaling=2,
    col = Col,
    pch = Symb,
    cex=1.5)


library(indicspecies)
```

```
library(multtest)
TarInd <- multipatt(Bac, Env$Treat, control = how(nperm=999))
summary(TarInd, indvalcomp = TRUE)


# ALPHA DIVERSITY


Env$ShanBac <- diversity(Bacteria, index = "shannon")
Env$ShanAlg <- diversity(Algae, index = "shannon")
Env$ShanFun <- diversity(Fungi, index = "shannon")

boxplot(NBacteria ~ Treat, data = Env)
boxplot(NAlgae ~ Treat, data = Env)
boxplot(NFungi ~ Treat, data = Env)

boxplot(ShanBac ~ Treat, data = Env)
boxplot(ShanAlg ~ Treat, data = Env)
boxplot(ShanFun ~ Treat, data = Env)

library(multcomp)
Env$Treat <- factor(Env$Treat)

NB <- glm(NBacteria ~ Treat, data = Env, family = quasipoisson)
summary(NB)
NB <- glm(NBacteria ~ Treat, data = Env, family = poisson)
anova(NB, test="Chisq")
PHNB <- glht(NB, linfct=mcp(Treat="Tukey"))
summary(PHNB)

NAl <- glm(NAlgae ~ Treat, data = Env, family = quasipoisson)
summary(NAl)
NAl <- glm(NAlgae ~ Treat, data = Env, family = poisson)
anova(NAl, test="Chisq")
PHNAl <- glht(NAl, linfct=mcp(Treat="Tukey"))
summary(PHNAl)

NF <- glm(NFungi ~ Treat, data = Env, family = quasipoisson)
summary(NF)
NF <- glm(NFungi ~ Treat, data = Env, family = poisson)
anova(NF, test="Chisq")
PHNF <- glht(NF, linfct=mcp(Treat="Tukey"))
```

```
summary(PHNF)

library(lmPerm)
NSB <- lm(ShanBac ~ Treat, data = Env)
anova(NSB, test="F")
NSBP <- lmp(ShanBac ~ Treat, data = Env)
anova.lmp(NSBP)
PHNSB <- glht(NSB, linfct=mcp(Treat="Tukey"))
summary(PHNSB)

NSA <- lm(ShanAlg ~ Treat, data = Env)
anova(NSA, test="F")
NSAP <- lmp(ShanAlg ~ Treat, data = Env)
anova.lmp(NSAP)
PHNSA <- glht(NSA, linfct=mcp(Treat="Tukey"))
summary(PHNSA)

NSF <- lm(ShanFun ~ Treat, data = Env)
anova(NSF, test="F")
NSFP <- lmp(ShanFun ~ Treat, data = Env)
anova.lmp(NSFP)
PHNSF <- glht(NSF, linfct=mcp(Treat="Tukey"))
summary(PHNSF)
```
